# Comparative Study of the Healing Process of Disbudding Wounds in Calves Using Bepanthene^®^ or an Antibiotic Spray

**DOI:** 10.3390/ani14172526

**Published:** 2024-08-30

**Authors:** Gabriela Martins, George Stilwell

**Affiliations:** 1 Animal Behaviour and Welfare Research Lab, Centre for Interdisciplinary Research in Animal Health, Faculty of Veterinary Medicine, University of Lisbon, Av. Universidade Técnica, Alto da Ajuda, 1330-477 Lisboa, Portugal; gabrielam@campus.ul.pt; 2 Associate Laboratory for Animal and Veterinary Sciences (AL4AnimalS), Faculty of Veterinary Medicine, University of Lisbon, Av. Universidade Técnica, Alto da Ajuda, 1300-477 Lisboa, Portugal

**Keywords:** disbudding, calves, healing, dexpanthenol, antimicrobial resistance

## Abstract

**Simple Summary:**

Antimicrobial resistance is seen as a global threat to One Health, since it is associated with high rates of morbidity and mortality. To reduce the use of antimicrobials in farm animals, Bepanthene® was compared with an antibiotic-based spray in the healing of disbudding wounds in calves. Benpanthene® is based on dexpanthenol, a derivative of pantothenic acid, also known as provitamin B5, which protects and stimulates skin regeneration. The findings of the present study support the use of Bepanthene®, allowing for a reduction in the use of antimicrobials in production animals without impeding healing.

**Abstract:**

The process of disbudding female calves is a common procedure in many dairy farms, avoiding injuries caused by horns and reducing feed bunk space requirements. The most common method for disbudding calves is by the use of a cautery iron, responsible for destroying the horn-generating tissue. After the procedure, wounds may be treated with an antibiotic-based spray. Nowadays, antimicrobial resistance is a worldwide concern in both human and veterinary medicine, highlighting the need to invest in the monitoring of antimicrobial use and in the development of alternative treatments in favour of One Health. The goal of this study is to promote a reduction in the use of antibiotics in farm animals by investigating an alternative treatment for disbudding wounds. Bepanthene^®^ (dexpanthenol, a derivative of pantothenic acid, a component of the B vitamin complex) is a plausible option, since it is widely used in human medicine for the treatment of skin irritations and burns. The comparison of the healing process of disbudding wounds treated with Bepanthene^®^ or a chlortetracycline-based spray was achieved through the presentation of a randomly-ordered sequence of images of the lesions to a panel of convenience-selected and blinded-to-treatment evaluators, composed of seven veterinarian practitioners, five veterinary medicine students, and five human medical field nurses. In order to classify the lesions, the panel applied an adapted format of a validated healing scale (Bates–Jensen Wound Assessment Tool), incorporating seven parameters of evaluation, culminating in the values used for statistical analyses. In the practitioners’ evaluation, a statistically significant effect for the factors of time and treatment in favour of Bepanthene^®^ was found for the parameters “Edges”, “Necrotic Tissue Type”, and “Skin Colour Surrounding Wound”, indicating that Bepanthene^®^ is superior to the spray when considering these parameters of healing. The assessment by the veterinary students showed a significant effect for the factors of time and treatment for the parameters “Necrotic Tissue Type”, in favour of the Bepanthene^®^, and “Granulation Tissue”, in favour of the antibiotic spray, demonstrating no clear benefit for either treatment. Lastly, the evaluation performed by nurses showed a significant effect for the factors of time and treatment, in favour of the Bepanthene^®^, for the parameters “Necrotic Tissue Type” and “Skin Colour Surrounding Wound”, leading to the conclusion that Bepanthene^®^ is associated with better and faster healing when compared to the spray. Overall, these findings lead us to suggest that Bepanthene^®^ presents a better healing index compared to a chlortetracycline-based spray, allowing it to be safely used as a substitute to an antimicrobial agent.

## 1. Introduction

The prevention of horn growth (disbudding) in female calves is a common practice on many dairy farms, since animals subjected to these procedures tend to be easier to handle, require less housing space, and are less prone to cause injuries to others [1]. The adequate age for disbudding varies considering the breed and method selected for disbudding [2].

For adequate pain management, disbudding should only be performed under local anaesthesia, and parenteral analgesics should be administered [3]. The process of disbudding, using chemicals or a heated cautery, is responsible for a rise in plasma cortisol and the display of pain-related behaviours, such as head shaking and rubbing, ear flicking, decreased grooming, restlessness, a decrease in rumination, and an increase in hind-leg kicks [4,5,6]. Usually, a single dose of parenteral analgesics is administrated on the day of the disbudding process, before the procedure. However, research conducted recently suggests that the administration of an additional dose of non-steroidal anti-inflammatory drugs (NSAIDs) three days following the procedure can cause a decline in pain-related behaviours in the one to two weeks following the disbudding process. However, this may also have negative implications, like slowing down the healing process of wounds [7].

The disbudding process can be accomplished through different methods, such as the use of thermocautery or a caustic paste. Thermocautery disbudding is achieved by applying a heated iron to the base of the horn bud to destroy the horn-generating tissue, causing deep burn wounds [8]. To prevent infection, protect the wound, and promote healing, an antibiotic-based product is sometimes sprayed on the wounds. However, a recent study highlights the absence of substantial differences between the healing of wounds treated with oxytetracycline aerosol spray and untreated wounds, leading to the conclusion that the use of antibiotic-based spray may not be needed for the healing of these lesions [9]. However, non-protected wounds attract flies, and the risk of infection is higher when environmental conditions are not well controlled.

Wound healing is responsible for restoring the normal anatomy and functionality of the injured tissue [10]. The wound healing process is divided into three distinct phases: inflammation, repair, and maturation. Initially, temporary vasoconstriction limits the haemorrhage, but later, vasodilation allows for the leaking of fibrinogen and clotting agents from the wound [11]. The triggering of coagulation mechanisms promotes platelet aggregation and adhesion, leading to the formation of a blood clot that provides haemostasis [12]. In the inflammatory phase, leucocytes and other cells migrate to the wound. Neutrophils are responsible for the debridement of the wound [11,13], while monocytes can turn into macrophages, responsible for the phagocytosis of debris and microorganisms and also for healing stimulation [14]. The granulation tissue, formed during the repair phase, is responsible for the protection of the wound and for filling defects [11,15]. The appearance of the tissue changes from a bright red, due to higher vascularization, to a paler presentation over time [16]. Fibroplasia is characterized by the migration and proliferation of fibroblasts [14,17], responsible for the production of fibrous tissue but also for removing damaged proteins [11,12,15]. Angiogenesis, stimulated by growth factors, is the process of neovascularization, beginning with the migration of endothelial cells to the wound, establishing a barrier against external infection and internal fluid loss [12,15]. The process of wound contraction, involving myofibroblasts, allows for a decrease in wound size [16], halting when the edges of the wound meet, when there is extreme tension, in the presence of necrotic tissue, or when myofibroblasts are deficient [18]. The maturation phase is characterized by the adequate deposition and strengthening of collagen, achieved with the alteration of the fibres’ orientation and increased crosslinking [11,14]. Healing may be delayed by the presence of bone sequestrum (a devitalized, and most often infected, piece of bone that acts as a foreign body). Bone sequestration has been identified in calves after disbudding [19]. Although burn wounds undergo the same healing process as other injuries, these types of wounds present an increase in the susceptibility to develop infections, which may result in sepsis and the aggravation of inflammation [20]. Burn wounds present a zone where protein denaturation, degradation, and coagulation occur and a second zone characterized by areas of ischemia and hypoxia, both resulting in tissue necrosis [20].

Nowadays, antimicrobial resistance has become a worldwide challenge, since it is associated with high rates of morbidity and mortality [21]. The difficulty in mitigating antimicrobial resistance lies somewhat in the abuse or improper use of antimicrobial drugs and the lack of information concerning this important topic. Broad-spectrum antibiotics continue to be recklessly used in unnecessary situations, contributing to this growing problem, especially at a time when new treatments for bacterial infections are limited [20]. Studies have shown that the indiscriminate use of topical agents containing antibiotics is related to the development and spread of bacterial resistance through the dissemination of resistance mechanisms across bacterial species [22]. The use of topical antibiotics has also been linked to a delay in the process of wound healing when compared with treatment using a topical gel containing cetrimide as well as local anaesthetics [23]. In this sense, there is a need to invest in the monitoring of antibiotics, new policies regulating their use, and new treatment alternatives to the use of antibiotics [24].

Considering these facts, the main objective of this study is to promote a reduction in the unnecessary use of antimicrobials in farm animals by investigating the value of an alternative treatment for disbudding wounds. Dexpanthenol (Bepanthene^®^) was the product considered as a substitute to the conventional antibiotic-based spray (chlortetracycline) used on a large number of farms following disbudding. Dexpanthenol is a derivative of pantothenic acid, a component of the B vitamin complex and an essential component of a normally functioning epithelium. Bepanthene^®^ is widely used in human medicine for the treatment of burns and skin irritations, promoting cellular division and migration, lipid production, and tissue growth and reducing inflammation [25,26]. Considering the lack of benefits associated with the use of an antibiotic-based spray on disbudding wounds [9] and the substantial use of Bepanthene^®^ as a healing agent in human medicine, it was expected that Bepanthene^®^ would present better results in comparison to the chlortetracycline spray in promoting healing of disbudding wounds.

## 2. Materials and Methods

All procedures were carried out with the approval of the Ethics Committee (Comissão de Ética para a Investigação e Ensino—CEIE) of the Faculty of Veterinary Medicine, University of Lisbon. The committee recognized the safeguarding of all ethical principles, approving the execution of the experimental protocol.

### 2.1. Farm and Animal Recruitment

The study was carried out on a dairy farm north of Lisbon, milking approximately 850 Holstein–Frisian cows. Calves are separated from the dam immediately after birth, kept in individual straw-bedded boxes (2 × 1 m) in an open-side barn, and fed by teat-bucket until they are approximately three weeks old. They are then transferred to an indoor straw-bedded group pen (20 × 10 m) with an automatic milking feeder. The calves’ first meal consists of three litres of pasteurized colostrum, administered using a feeding tube as soon as possible following birth. When in the individual crates, the animals are fed three litres of whole milk two times a day, and once they are transferred to the indoor group pen, the milk is distributed using an automated milk feeder, with calves drinking a maximum of six to seven litres per day. The quantity of ingested milk then decreases gradually until weaning. The pure Holstein–Friesian female calves remain on the farm with the intention of breeding, while the male dairy calves and crosses with beef breeds are sold when approximately 15 days old.

There were no particular criteria in the selection of the animals since all Holstein female calves undergo the same disbudding procedure at three weeks of age and all animals presented for disbudding during the experimental periods were included in this study. A total of 27 animals were used for this study, divided into two groups based on when the routine disbudding was performed (Table 1). The sample size was dependent on the visits to the farm, the number of animals available for disbudding at each visit, and timeframes related to the completion of this study. All calves were Holstein–Friesian females approximately three weeks old (+/−3 days).

### 2.2. Disbudding and Treatments

The process of disbudding started with the immobilization of the animals to facilitate the procedure and avoid accidents that may result in injuries. The individual boxes were equipped with a closing head lock that allowed for easy access to the animal’s head outside of the box, placing pressure on the neck and preventing the calf from moving backwards, thereby allowing the realization of the procedure. Additionally, a person inside the box gently pressed the animal forward, avoiding backing up during the procedure.

After the animal was properly restrained, local anaesthesia and sub-cutaneous analgesia were administered using a 20-gauge needle. The cornual nerve block was achieved by injecting 6 mL of procaine (Procamidor^®^ 20 mg/mL) midway between the horn bud and the medial canthus of the eye (dorsal to the supra-orbital foramen) on each side. Effective nerve block administration was checked by pricking the bud area with a needle before proceeding with the disbudding. Regarding analgesia, each animal received 1.5 mL of caprofen (Carprosan^®^ 50 mg/mL, Dechra; approximately 1.4 mg per kg bodyweight or 1 mL/kg) subcutaneously. Standard clippers were used to clip the hair, starting by identifying the horn and cutting the hair around it, leaving a margin superior to two centimetres from the edges of the horn bud.

The process of disbudding started approximately ten minutes after the administration of the drugs using an electric thermocautery. The hot iron was applied with a downward force against the skull of the animal, with a rotating motion in order to cut along the base of the horn bud. As soon as the skin detaches from the underlying tissue, the iron is placed obliquely and forced upwards to remove the entire horn bud. The procedure culminates with two circular burn wounds, on each side of the skull. The wounds were then treated with the two products: Bepanthene^®^, an amount roughly the size of a hazelnut, was applied using a gloved finger to the wound on the right side of the skull, while the chlortetracycline-based spray (Cyclospray^®^) was used to treat the wound on the left side. Treatment was applied only once, as is usually performed on commercial farms. The application of Bepanthene^®^ was performed under the cascade, as it is a widely used product in humans and recommended withdrawal intervals for meat and milk are easily applied in these young calves.

A non-treated control group was not included, as the farmer did not accept an unprotected wound for fear of flies’ harassment or infection.

### 2.3. Assessment of the Disbudding Wounds

The first group (*N* = 13) was disbudded on the 20th of October, while the second group (*N* = 14) underwent the procedure on the 17th of November. Periodic assessments were made for both groups, establishing strict and similar time intervals for the evaluations (Table 1).

The selection of the time intervals for the periodic assessments was performed considering the timeframe for the realization of the study and the availability of the authors to visit the farm. At these times, pictures of both wounds were taken at the same distance (approximately 10 centimetres) and with the same camera (iPhone 11^®^, 12 MP-resolution). To obtain photographic evidence of the injuries, calves were gently restrained by one person while the other took the photographs. Subsequently, 430 pictures (27 calves × 2 wounds × 8 assessment moments; minus 2 images because of a dead calf as described in Table 1) were uploaded to a computer and used to create a PowerPoint presentation where each photograph was given a specific code and displayed randomly in order to avoid a biased classification. The evaluation of the healing process was conduction by using a validated healing scale designated the “Bates–Jensen Wound Assessment Tool” [27]. However, for this particular study, the scale was modified to fit the requirements of the trial by eliminating parameters impossible to evaluate through images. Therefore, our adapted Bates–Jensen Wound Assessment Tool only included seven parameters: edges, necrotic tissue type, exudate type, exudate amount, skin colour surrounding the wound, granulation tissue, and epithelialization (Appendix A). The parameters were evaluated on a scale of one to five, one being the best score and five being the worst.

There was also the possibility of using “NA” for “Not Applicable” for dubious situations or when the parameter of classification could not be seen. To allow for statistical analysis, this option received a neutral score of 3.

Once the wound was rated for all the parameters, the total score was determined by adding all of the scores. The higher the total score, the more severe the wound status [27].

To obtain a wide-ranging and representative evaluation of the lesions, a panel of blinded-to-treatment evaluators was created, composed of seven experienced practitioners, five veterinary medicine students, and five experienced nurses from the human medical field. Each of the evaluators was tasked with the classification of the 430 pictures, considering the chosen healing scale. Assessors were previously presented the healing scale descriptions with examples so as to be familiar with the tool and its different parameters. Although assessors were asked to disregard it, evidence of the spray was present in some of the early photographs, so a fully blinded evaluation could not be ensured. The amount and complexity of the work required was very significant, explaining why we did not receive a response from some of the invited evaluators (in total, we invited 7 individuals in each group).

### 2.4. Statistical Analysis

The statistical analysis was conducted using both Microsoft Excel^®^ 2013 (Seattle, WA, USA) for the organization of data and GraphPad Prism 9 software (La Jolla, CA, USA), specifically for the design of graphics and statistical analysis. Before conducting any tests, for all continuous variables, an assumption of normality was evaluated by the Shapiro–Wilks and Kolmogorov–Smirnov statistical tests, taking into consideration the respective measures of skewness and kurtosis and visualizing histogram normal distributions. To determine if the difference between Bepanthene^®^ and the antibiotic spray was statistically significant, a two-way ANOVA was used, comparing the means of classifications given to each parameter in the four time intervals. To further evaluate the nature of the differences between the two treatments applied, multiple comparisons post-hoc tests were performed, such as Tukey’s HSD post-hoc test or Bonferroni’s test. The distribution of the sample values for each of the treatments and individual parameters was assessed using a two-way ANOVA. All tests were performed with a 95% confidence interval, and statistical significance was recognized when the *p* value was ≤ 0.05. Lastly, the ROUT method of identifying outliers was applied using the GraphPad Prism software to detect outliers among any sample, which could later be removed from the statistical analyses. In this particular analysis, there were no outliers detected among the sample values.

All assessments made by the classifiers were organized and statistically analysed by the first author of this study.

## 3. Results

### 3.1. Panel 1—Veterinary Practitioners

Seven experienced practitioners were asked to evaluate the images, and all responded back. This sub-panel showed the most extensive range of values for most of the parameters as a result of an unbalanced and uneven evaluation, sometimes showing little coherence between the classifications. However, a statistical analysis of the obtained data revealed that for the parameter “Edges”, there was an effect (*p* < 0.05) for the factors of time (days) and treatment in favour of Bepanthene^®^ but no effect for the interaction between the two factors (Table 2). While the two-way ANOVA showed a difference between the two products, the multiple comparisons test did not find a significant difference between the treatments on the different time intervals. For the parameter “Necrotic Tissue Type”, there was an effect (*p* < 0.05) for the factors of time (days) and treatment in favour of Bepanthene^®^ but no effect for the interaction between the two factors (Table 2). For this parameter, considering the results obtained in the multiple comparisons test, a difference was found between Bepanthene^®^ and the spray in the time intervals of two (*p* = 0.009), five (*p* = 0.0374), and twenty days (*p* = 0.0438), favouring the treatment with Bepanthene^®^. Lastly, for the parameter “Skin Colour Surrounding Wound”, there was an effect (*p* < 0.05) for the factors of time (days) and treatment in favour of Bepanthene^®^ but no effect for the interaction between the two factors (Table 2). Considering the multiple comparisons test, this parameter presented a difference between the two products on the fifth day (*p* < 0.0001) following the procedure, favouring Bepanthene^®^. Regarding the remaining parameters, there was no significant difference between treatments.

### 3.2. Panel 2—Veterinary Medicine Students

Five fifth-year veterinary medicine students were asked to evaluate the 432 collected images. It was possible to verify that the veterinary students possessed the least extensive range of values for the evaluations, indicating a very homogenous classification. A statistical analysis of the obtained data (Table 3) revealed that for the parameter “Necrotic Tissue Type”, there was a significant effect (*p* < 0.05) for the factors of time (days) and treatment favouring Bepanthene^®^ but no effect for the interaction between the two factors (Table 3). In this case, considering the multiple comparisons test, a difference was present between the used treatments, Bepanthene^®^ and spray, on the fifth day (*p* < 0.0001) following the procedure, favouring the use of Bepanthene^®^. Lastly, the parameter “Granulation Tissue” presented a significant effect (*p* < 0.05) for the factors of time (days) and treatment favouring the antibiotic-based spray but no effect for the interaction between the two factors (Table 3). The multiple comparisons test displayed a significant difference between the two products on the fifth day (*p* = 0.0447) following disbudding, favouring the use of the antibiotic-based spray. Regarding the remaining parameters, no difference between treatments was shown.

### 3.3. Panel 3—Nurses from the Human Medical Field

Five experienced nurses were asked to evaluate the collected images. It was possible to verify that the nurses possessed quite an extensive range of values, resulting from an unbalanced and uneven classification exhibiting little coherence between classifications. For the parameter “Necrotic Tissue Type”, there was an effect (*p* < 0.05) for the factors of time (days) and treatment in favour of Bepanthene^®^ but no effect for the interaction between the two factors (Table 4). Considering the multiple comparisons test, this parameter presented a difference between the two products on the fifth day (*p* = 0.0251) following the procedure, favouring Bepanthene^®^. Lastly, concerning the parameter “Skin Colour Surrounding Wound”, a significant effect (*p* < 0.05) for the factors of time (days) and treatment in favour of Bepanthene^®^ but no effect for the interaction between the two factors (Table 4) was shown. The multiple comparisons test displayed a difference between the two products on the fifth day (*p* = 0.0468) following disbudding, favouring the use of the alternative treatment, Bepanthene^®^. Regarding the remaining parameters, no statistically significant difference between treatments was found.

### 3.4. Joint Evaluation

In order to develop an overall perception of the scoring, the average values of the scores attributed by the evaluators in each panel were calculated. Table 5 summarizes the results obtained for this all-panel classification. It is evident that Bepanthene^®^-treated wounds had lower values compared to the spray in all parameters except for “Granulation Tissue”. However, a statistically significant difference was only found for the parameters “Edges” and “Necrotic Tissue Type” (*p* = 0.0001 and *p* = 0.0064, respectively).

#### “Not Applicable” Responses

The classification “NA”, absent in the original healing scale, was considered an intermediate classification; in other words, a neutral score of three was given. This was a way of avoiding the use of zero, which would impair statistical analysis. The use of an intermediate rating is liable to cause some deviation in the results since the rating can be over- or undervalued. The group of evaluators that used “NA” most often was the veterinary students, followed by the nurses. The use of this classification was more frequent in the pictures that corresponded to wounds treated with Cyclospray^®^.

## 4. Discussion

Considering the rise in antimicrobial resistance, in a small part probably associated with the use of topical agents containing antibiotics [22], the need to develop alternative treatments has been rapidly increasing. Previous studies have shown that the application of an antibiotic-based spray, oxytetracycline aerosol spray, to disbudding wounds presents no benefits in comparison to untreated wounds, negating the need to use this product on these types of lesions [9]. However, farmers still prefer to cover the wound after disbudding, and no study has looked at the healing of non-treated wounds on commercial farms in hot countries where flies are abundant (even in autumn) and rapidly envelop fresh wounds, causing very poor animal welfare. For these reasons, offering farmers an alternative to antibiotic spray treatment is essential.

The main objective of this study was to assess the possibility of replacing the antibiotic-based spray with an alternative product, Bepanthene^®^, widely used in human medicine for the treatment of burns and skin irritations [26]. This paste can be used under the cascade without any inconvenience, as required withdrawal periods are very easily fulfilled in these young calves (7 days for milk and 28 days for meat). The viability of this hypothesis was determined by comparing the healing processes of disbudding wounds using Bepanthene^®^ or Cyclospray^®^, a chlortetracycline-based spray. As was described in the Material and Methods, having an untreated wound was not acceptable, as the welfare of these animals could be very poor due to harassment by flies. Also, the farmer would not accept having exposed wounds for fear of infection and fly maggots.

All animals were of the same age and born on the same farm, which means they were subjected to the same husbandry conditions. The procedure was carried out as usually performed on commercial farms. Sedation (e.g., xylazine) was not used, as it carries some danger, and when local anaesthesia is well applied, there is no struggling, and handling is very easy.

The use of a pre-existing, complex, and validated healing scale, the Bates–Jensen Wound Assessment Tool [27], was also an advantage since it has been successfully and widely used in the determination of wound status. The process of disbudding was accomplished using a hot iron, as this is the method, according to some studies, that allows for better results and more efficient acute pain management in comparison to other techniques [3,28]. However, this method does not guarantee total safeguarding of the calves’ welfare, since the wounds probably remain painful throughout the nine-week healing period [29]. In our study, the disbudding process was accompanied by proper analgesia and local anaesthesia [30], ensuring the welfare of the animals. Nonetheless, in a future replication of this study, the administration of an additional dose of NSAIDs three days following the procedure is advised, since recent studies suggest the connection to a decrease in pain-related behaviours [7]. Lastly, the attainment of photographic evidence was performed by the same person, ensuring consistency. For the duration of the study, there were no evident complications such as exuberant infection of the wounds, loss of appetite, or abnormal signs of severe discomfort related to the procedure or the subsequent healing process. However, at the final assessment of the second group, it was noted that one of the animals had died due to illness (pneumonia) unrelated to the process of disbudding (Table 1).

The evaluation of disbudding wound healing was accomplished through the observation of images of the injuries and using the Bates–Jensen Wound Assessment Tool for grading. With this tool, a higher score corresponds to a more severe wound status. The assessment was performed by a panel of blinded-to-treatment evaluators, constituting seven experienced practitioners, five veterinary medicine students, and five experienced nurses from the human medical field. Following the organization of the collected data, it was possible to affirm that the veterinarians possessed the most extensive range of values for the evaluations, while the veterinary students presented the least extensive range of values for the evaluation. The composition of the groups may be a factor that contributed to the discrepancies within the group, since men and women tend to have different perspectives of pain and injury severity [31]. Five men and two women constituted the veterinarian panel; the nurse panel was made up of two men and three women; and lastly, the student panel integrated one man and four women. The students presented more uniformity in the composition of the panel, which may contribute to the uniformity of the evaluations. Lastly, the differences may be related to the attention paid to detail, since practitioners are more used to evaluating disbudding wounds, paying more attention to details that the untrained eye may not capture, and recognizing different patterns related to wound infection in calves.

When considering the practitioners’ panel analysis, an effect for the factors of time and treatment in favour of the Bepanthene^®^ was found for the parameters “Edges”, “Necrotic Tissue Type”, and “Skin Colour Surrounding Wound”. However, the multiple comparisons test only found differences between the two products for the parameter “Necrotic Tissue Type” in the time intervals of two, five, and twenty days and for “Skin Colour Surrounding Wound” on day five. However, the statistically significant difference present in the two-way ANOVA analyses still suggests the superiority of Bepanthene^®^ for the healing of disbudding wounds. These findings lead us to conclude that, according to the practitioners’ evaluation, Bepanthene^®^ is associated with better and faster healing, or at least shows potential for the sound healing of disbudding wounds.

In the students’ panel, an effect for the factors of time and treatment was found for the parameter “Necrotic Tissue Type” in favour of Bepanthene^®^ and for “Granulation Tissue” in favour of the spray. The multiple comparisons test presented, a significant difference in favour of the Bepanthene^®^ for the parameter “Necrotic tissue Type” on day five following the procedure and a significant difference in favour of the spray for the parameter “Granulation Tissue” on day five. In light of these results, it is possible to conclude that the veterinary students’ results do not statistically support any of the products. However, it should be said that the raw values presented for Bepanthene^®^ were almost always lower than the spray’s values.

Lastly, in the nurses’ panel, an effect for the factors of time and treatment in favour of the Bepanthene^®^ was found for the parameters “Necrotic Tissue Type” and “Skin Colour Surrounding Wound”. The multiple comparisons test presented a significant difference between the two products in the five days following the procedure for both the parameters “Necrotic Tissue Type” and “Skin Colour Surrounding Wound”. These findings lead to the conclusion that, according to the nurses’ scrutiny, Bepanthene^®^ is associated with better and faster healing, or at least shows potential for the improved healing of disbudding wounds, when considering the parameters previously mentioned.

The study presented some limitations that must be taken into consideration when analysing the results. Considering the materials and methods, the use of different treatments on each side of the animal presents a reasonable limitation, since studies show that disbudding wounds present on the left side of the skull tend to re-epithelialize faster, present a shorter crust period and, considering the appearance, are cooler and smaller than right side lesions [32]. It is suggested that in further studies, the side for each treatment should be randomly selected.

One of the most noticeable limitations, pointed out by various participants, was the classification complexity. The PowerPoint presentation contained 430 pictures, requiring extensive hours to assess all cases. Another problem was the fact that some of the wounds treated with Cyclospray^®^ presented a slight blue tint due to the colouring agent present in the product, making it difficult to evaluate parameters related to the observation of colour in the first days. In response to this problem, the assessors may have resorted to the use of the “Not Applicable” rating, creating further limitations to the study. The use of an intermediate and neutral score for the NA classification aids in the decrease in standard deviations, favouring the statistical analyses, but it does not solve the problem, since some parameters may have been over- or undervalued, influencing the overall classification. Variations throughout the evaluation were evident. At the beginning of the classification, the use of the classification “Not Applicable” was more prominent when compared to the end of the classification, indicating that the participants started to become more comfortable with the process as time went by.

Another problem was the ease in the distinction of the product used for the treatment of the wounds in the first evaluation. Despite asking the participants to ignore the blue colouring agent characteristic of the Cyclospray^®^, more than half of the fifth-day wound pictures presented some blue colouration. This may have influenced the classifier, even if unwittingly.

Considering the photographs, there were some differences in light exposure due to the periodic routine movement of animals between pens that had different lighting conditions. Additionally, as animals grew, restraint and immobilization became more difficult. However, the impact of these variations was small and common to all groups.

Lastly, the population size of 27 animals, due to availability and scheduling, was perhaps small for the study. The program G*Power (Germany), considering a two-tailed paired *t*-test and assuming an effect size of 0.5 and a power of 0.95, determined an ideal sample size of 54 animals [23]. However, because the study was conducted on a commercial farm, it was not easy to obtain the desired sample size at the right moments. We recall that all calves available for disbudding during the study period were used.

Although there is strong evidence that Bepanthene^®^ does improve healing when compared with an antibiotic-based spray, it is suggested to repeat the study considering some solutions for the various problems encountered. For example, the evaluators must be given the necessary time to present their classifications, without being rushed; the spray-based antibiotic must be replaced by another product without the colouring agent or proceed with the removal of the blue colour upon the first re-evaluation to avoid the wound parameters being masked; the possibility of the classification “Not Applicable” should be eliminated to obtain more consistent and reliable results; the evaluators should be advised to study well the healing scale used, as well as the images that will be evaluated, in order to be familiarized with the process of classification before it starts in hopes of avoiding the learning curve presented throughout the evaluation; when taking the pictures used for the presentation, similar conditions of lighting must be ensured, and the animals must be properly restrained; and lastly, the sample size should be increased to the value presented previously in order to obtain more robust results.

Finally, a word on the potential use of Benpanthene on food-producing animals: Although these calves will not produce milk or be slaughtered for meat in the following couple of years, and Benpathene^®^ is considered safe to be applied to humans (even in small children), the cascade should be used until studies on residues are published. This means that a 7-day withdrawal interval for milk and a 28-day withdrawal for meat should be imposed.

## 5. Conclusions

Offering farmers an alternative to antibiotic spray to cover fresh disbudding wounds in commercial settings is important. Although the evidence presented in this study is based on a subjective assessment of healing pictures, demonstrating some differences between evaluators, it does suggest that the treatment of disbudding wounds with Bepanthene^®^ is a suitable replacement for antibiotic-based sprays, since it presented an overall better healing index across all panels. So it is suggested that this product can be safely used as a substitute when disbudding young calves. The use of Bepanthene^®^ will not only allow for better healing of the injuries resulting from cautery disbudding but will also help in achieving a reduction in the use of topical antibiotics in farm animals.

## Figures and Tables

**Table 1:** Time intervals for the assessments of each group since the day of disbudding.

Groups	Day of Assessment	Days from Disbudding
Group 1 (13 animals)	20 October 2021	Day of disbudding
	22 October 2021	Two days
	25 October 2021	Five days
	9 November 2021	Twenty days
	19 November 2021	Thirty days
Group 2 (14 animals)	17 November 2021	Day of disbudding
	19 November 2021	Two days
	22 November 2021	Five days
	7 December 2021	Twenty days
	17 December 2021	Thirty days *

* At this moment only 13 animals were assessed, as one had died from respiratory disease.

**Table 2:** Relationship between Bepanthene^®^ and antibiotic spray for each parameter in the practitioners’ evaluation. Scoring scale from 1 (no evidence) to 5 (very evident) (Appendix A).

Pt	Time	Bepanthene	Cyclospray	Significance
Edges	2 days	3.19 ± 0.39	3.34 ± 0.28	Time effect: F (1.976, 102.1) = 24.15, *p* < 0.01 * Treatment effect: F (1, 52) = 7.40, *p* = 0.01 * Interaction time–treatment: F (3, 155) = 0.47, *p* = 0.71
	5 days	3.10 ± 0.46	3.35 ± 0.48	
	20 days	3.00 ± 0.74	3.20 ± 0.58	
	30 days	2.10 ± 0.97	2.53 ± 1.05	
Necrotic Tissue	2 days	2.38 ± 0.42	2.77 ± 0.47	Time effect: F (2.249, 117.0) = 18.28, *p* < 0.01 * Treatment effect: F (1, 52) = 19.13, *p* < 0.01 * Interaction time–treatment: F (3, 156) = 0.05, *p* = 0.98
	5 days	2.63 ± 0.48	2.98 ± 0.45	
	20 days	2.60 ± 0.41	2.97 ± 0.58	
	30 days	1.94 ± 0.74	2.25 ± 0.84	
Exudate Type	2 days	1.52 ± 0.66	1.50 ± 0.85	Time effect: F (2.429, 126.3) = 3.11, *p* < 0.04 * Treatment effect: F (1, 52) = 0.38, *p* = 0.54 Interaction time–treatment: F (3, 156) = 0.3, *p* = 0.15
	5 days	1.48 ± 0.41	1.50 ± 0.81	
	20 days	1.77 ± 0.98	1.55 ± 0.90	
	30 days	1.26 ± 0.36	1.24 ± 0.33	
Exudate Amount	2 days	1.30 ± 0.42	1.33 ± 0.59	Time effect: F (2.422, 125.9) = 2.41, *p* = 0.08 Treatment effect: F (1, 52) = 0.09, *p* = 0.76 Interaction time–treatment: F (3, 156) = 0.63, *p* = 0.59
	5 days	1.22 ± 0.23	1.31 ± 0.52	
	20 days	1.50 ± 0.74	1.34 ± 0.58	
	30 days	1.20 ± 0.33	1.15 ± 0.26	
Skin Colour	2 days	2.51 ± 0.37	2.80 ± 0.49	Time effect: F (2.776, 144.4) = 22.61, *p* < 0.01 * Treatment effect: F (1, 52) = 13.75, *p* = 0.01 * Interaction time–treatment: F (3, 156) = 1.53, *p* = 0.21
	5 days	2.61 ± 0.32	3.03 ± 0.32	
	20 days	2.60 ± 0.38	2.75 ± 0.38	
	30 days	2.13 ± 0.62	2.22 ± 0.54	
Granulation Tissue	2 days	3.87 ± 0.34	3.81 ± 0.31	Time effect: F (2.288, 119.0) = 52.40, *p* < 0.01 * Treatment effect: F (1, 52) = 0.11, *p* = 0.74 Interaction time–treatment: F (3, 156) = 0.28, *p* = 0.84
	5 days	3.81 ± 0.35	3.83 ± 0.41	
	20 days	3.55 ± 0.64	3.66 ± 0.45	
	30 days	2.80 ± 0.77	2.86 ± 0.67	
Epithelialization	2 days	4.38 ± 0.27	4.42 ± 0.33	Time effect: F (1.457, 75.77) = 38.41, *p* < 0.01 * Treatment effect: F (1, 52) = 0.91, *p* = 0.34 Interaction time–treatment: F (3, 156) = 0.03, *p* = 0.99
	5 days	4.33 ± 0.35	4.45 ± 0.27	
	20 days	4.22 ± 0.55	4.29 ± 0.46	
	30 days	3.25 ± 1.11	3.34 ± 1.02	

Legend: * Statistically significant difference (*p* value < 0.05) between the two products. The values of the classifications for each of the time intervals and respective parameters are presented as means ± standard deviation. The column “Significance” characterizes the significance between the two products, Bepanthene^®^ and Cyclospray^®^, presenting the degrees of freedom and the *p* values. Pt—Parameters.

**Table 3:** Relationship between Bepanthene^®^ and antibiotic spray for each parameter in the veterinary students’ evaluation. Scoring scale from 1 (no evidence) to 5 (very evident) (Appendix A).

Pt	Time	Bepanthene^®^	Spray	Significance
Edges	2 days	3.07 ± 0.55	3.24 ± 0.35	Time effect: F (2.147, 111.7) = 18.59, *p* < 0.01 * Treatment effect: F (1, 52) = 1.499, *p* = 0.23 Interaction time–treatment: F (3, 156) = 0.16, *p* = 0.92
	5 days	3.20 ± 0.38	3.29 ± 0.41	
	20 days	3.35 ± 1.05	3.40 ± 0.93	
	30 days	2.21 ± 1.07	2.46 ± 1.20	
Necrotic Tissue	2 days	2.31 ± 0.69	2.81 ± 0.54	Time effect: F (2.712, 141.0) = 14.20, *p* < 0.01 * Treatment effect: F (1, 52) = 27.71, *p* < 0.01 * Interaction time–treatment: F (3, 156) = 1.08, *p* = 0.36
	5 days	2.33 ± 0.38	3.00 ± 0.57	
	20 days	2.31 ± 0.37	2.62 ± 0.58	
	30 days	1.82 ± 0.54	2.17 ± 0.87	
Exudate Type	2 days	1.01 ± 0.07	1.21 ± 0.41	Time effect: F (1.980, 103.0) = 2.263, *p* = 0.11 Treatment effect: F (1, 52) = 0.74, *p* = 0.39 Interaction time–treatment: F (3, 156) = 0.87, *p* = 0.46
	5 days	1.03 ± 0.15	1.16 ± 0.63	
	20 days	1.34 ± 0.79	1.30 ± 0.78	
	30 days	1.20 ± 0.29	1.14 ± 0.30	
Exudate Amount	2 days	1.00 ± 0.03	1.18 ± 0.35	Time effect: F (2.028, 105.5) = 2.709, *p* = 0.07 Treatment effect: F (1, 52) = 1.916, *p* = 0.17 Interaction time–treatment: F (3, 156) = 0.75, *p* = 0.52
	5 days	1.01 ± 0.07	1.10 ± 0.40	
	20 days	1.24 ± 0.57	1.24 ± 0.55	
	30 days	1.11 ± 0.16	1.11 ± 0.25	
Skin Colour	2 days	1.45 ± 0.35	1.59 ± 0.46	Time effect: F (2.810, 146.1) = 1.79, *p* = 0.15 Treatment effect: F (1, 52) = 0.03, *p* = 0.86 Interaction time–treatment: F (3, 156) = 1.4, *p* = 0.24
	5 days	1.50 ± 0.46	1.47 ± 0.35	
	20 days	1.70 ± 0.37	1.57 ± 0.40	
	30 days	1.51 ± 0.42	1.59 ± 0.43	
Granulation Tissue	2 days	4.01 ± 0.49	3.71 ± 0.42	Time effect: F (2.595, 179.9) = 30.48, *p* < 0.01 * Treatment effect: F (1, 208) = 5.729, *p* = 0.02 * Interaction time–treatment: F (3, 208) = 0.51, *p* = 0.68
	5 days	4.05 ± 0.43	3.73 ± 0.45	
	20 days	3.85 ± 0.82	3.73 ± 0.61	
	30 days	2.95 ± 0.93	2.87 ± 0.61	
Epithelialization	2 days	4.18 ± 0.49	3.98 ± 0.38	Time effect: F (2.129, 110.7) = 28.74, *p* < 0.01 * Treatment effect: F (1, 52) = 0.02, *p* = 0.89 Interaction time–treatment: F (3, 156) = 1.14, *p* = 0.34
	5 days	4.01 ± 0.54	4.01 ± 0.39	
	20 days	3.91 ± 0.57	3.95 ± 0.45	
	30 days	3.07 ± 0.82	3.28 ± 0.89	

Legend: * Statistically significant difference (*p* value < 0.05) between the two products. The values of the classifications for each of the time intervals and respective parameters are presented as means ± standard deviation. The column “Significance” characterizes the significance between the two products, Bepanthene^®^ and antibiotic spray, presenting the degrees of freedom and the *p* values. Pt—Parameters.

**Table 4:** Relationship between Bepanthene^®^ and antibiotic spray for each parameter in the nurses’ evaluation. Scoring scale from 1 (no evidence) to 5 (very evident) (Appendix A).

Pt	Time	Bepanthene	Spray	Significance
Edges	2 days	3.05 ± 0.38	3.17 ± 0.35	Time effect: F (1.902, 131.9) = 29.23, *p* < 0.01 * Treatment effect: F (1, 208) = 3.72, *p* = 0.06 Interaction time–treatment: F (3, 208) = 0.15, *p* = 0.93
	5 days	3.02 ± 0.36	3.22 ± 0.42	
	20 days	2.88 ± 0.50	3.06 ± 0.49	
	30 days	2.24 ± 0.77	2.31 ± 0.81	
Necrotic Tissue	2 days	2.18 ± 0.60	2.41 ± 0.45	Time effect: F (2.553, 132.7) = 11.80, *p* < 0.01 * Treatment effect: F (1, 52) = 8.42, *p* < 0.01 * Interaction time–treatment: F (3, 156) = 0.52, *p* = 0.67
	5 days	2.07 ± 0.58	2.48 ± 0.45	
	20 days	2.08 ± 0.44	2.28 ± 0.47	
	30 days	1.68 ± 0.56	1.88 ± 0.74	
Exudate Type	2 days	1.17 ± 0.33	1.20 ± 0.57	Time effect: F (2.094, 108.9) = 2.161, *p* = 0.12 Treatment effect: F (1, 52) = 0.65, *p* = 0.42 Interaction time–treatment: F (3, 156) = 0.12, *p* = 0.95
	5 days	1.17 ± 0.36	1.31 ± 0.54	
	20 days	1.43 ± 0.83	1.45 ± 0.90	
	30 days	1.21 ± 0.31	1.28 ± 0.31	
Exudate Amount	2 days	1.17 ± 0.21	1.23 ± 0.38	Time effect: F (2.112, 109.8) = 3.05, *p* < 0.05 * Treatment effect: F (1, 52) = 0.61, *p* = 0.44 Interaction time–treatment: F (3, 156) = 0.14, *p* = 0.94
	5 days	1.20 ± 0.22	1.28 ± 0.29	
	20 days	1.42 ± 0.62	1.41 ± 0.51	
	30 days	1.25 ± 0.33	1.27 ± 0.31	
Skin Colour	2 days	2.17 ± 0.35	2.29 ± 0.44	Time effect: F (2.692, 186.7) = 22.73, *p* < 0.01 * Treatment effect: F (1, 208) = 0.02, *p* < 0.01 * Interaction time–treatment: F (3, 208) = 0.31, *p* = 0.82
	5 days	2.10 ± 0.38	2.38 ± 0.40	
	20 days	2.06 ± 0.53	2.25 ± 0.57	
	30 days	1.54 ± 0.44	1.70 ± 0.48	
Granulation Tissue	2 days	3.19 ± 0.39	3.00 ± 0.37	Time effect: F (2.761, 143.6) = 13.59, *p* < 0.01 * Treatment effect: F (1, 52) = 1.23, *p* = 0.27 Interaction time–treatment: F (3, 156) = 0.36, *p* = 0.78
	5 days	3.10 ± 0.44	3.04 ± 0.43	
	20 days	3.11 ± 0.61	3.05 ± 0.49	
	30 days	2.59 ± 0.59	2.60 ± 0.53	
Epithelialization	2 days	3.91 ± 0.47	3.83 ± 0.49	Time effect: F (2.479, 128.9) = 14.52, *p* < 0.01 * Treatment effect: F (1, 52) = 0.15, *p* = 0.69 Interaction time–treatment: F (3, 156) = 0.44, *p* = 0.73
	5 days	3.91 ± 0.57	3.80 ± 0.48	
	20 days	3.86 ± 0.70	3.77 ± 0.58	
	30 days	3.30 ± 0.88	3.38 ± 0.72	

Legend: * Statistically significant difference (*p* value < 0.05) between the two products. The values of the classifications for each of the time intervals and respective parameters are presented as means ± standard deviation. The column “Significance” characterizes the significance between the two products, Bepanthene^®^ and antibiotic spray, presenting the degrees of freedom and the *p* values. Pt—Parameters.

**Table 5:** Overall evaluation—values for each parameter are the average of scores by the three panels. Scoring scale from 1 (no evidence) to 5 (very evident) (Appendix A).

Parameters	Added Bepanthene Values	Added Spray Values	*p* Value
Edges	308.9 ± 63.87	327.8 ± 66.49	<0.0001
Necrotic Tissue Type	238.9 ± 67.59	276.5 ± 97.18	<0.0064
Exudate Type	142.5 ± 32.67	143.8 ± 31.86	0.33
Exudate Amount	132.2 ± 26.21	134.7 ± 30.59	0.156
Skin Colour Surrounding Wound	220.6 ± 79.77	235.8 ± 96.33	0.12
Granulation Tissue	368.4 ± 114.9	361.2 ± 107.2	0.84
Epithelialization	418.6 ± 73.56	420.9 ± 62.90	0.77

Legend: The values for each of the parameters are presented as means ± standard deviation. The column for the *p* value shows the significance between the two products, Bepanthene^®^ and antibiotic-based spray.

## Data Availability

The data presented in this study are available on request from the corresponding author.

## Appendix A. Adapted Bates–Jensen Wound Assessment Tool

**Item**	**Assessment**
Edges	1 = Indistinct, diffuse, none clearly visible 2 = Distinct, outline clearly visible, attached, even with wound base 3 = Well-defined, not attached to wound base 4 = Well-defined, not attached to base, rolled under, thickened 5 = Well-defined, fibrotic, scarred, or hyperkeratotic
Necrotic Tissue Type	1 = None visible 2 = White/grey non-viable tissue and/or non-adherent yellow slough 3 = Loosely adherent yellow slough 4 = Adherent, soft, black eschar 5 = Firmly adherent, hard, black eschar
Exudate Type	1 = None 2 = Bloody 3 = Serosanguineous: thin, watery, pale red/pink 4 = Serous: thin, watery, clear 5 = Purulent: thin or thick, opaque, tan/yellow
Exudate Amount	1 = None, dry wound 2 = Scant, wound moist but no observable exudate 3 = Small 4 = Moderate 5 = Large
Skin Colour Surrounding Wound	1 = Pink or normal for breed 2 = Bright red and/or congested 3 = White or grey pallor or hypopigmented 4 = Dark red or purple and/or haemorrhagic 5 = Black or hyperpigmented
Granulation Tissue	1 = Skin intact or partial-thickness wound 2 = Bright, beefy red; 75% to 100% of wound filled and/or tissue overgrowth 3 = Bright, beefy red; <75% and >25% of wound filled 4 = Pink and/or dull, dusky red and/or fills < 25% of wound 5 = No granulation tissue present
Epithelialization	1 = 100% wound covered, surface intact 2 = 75% to <100% wound covered and/or epithelial tissue extends >0.5cm into the wound bed 3 = 50% to <75% wound covered and/or epithelial tissue extends to <0.5 cm into the wound bed 4 = 25% to <50% wound covered 5 = <25% wound covered
